# Rolling Resistance Evaluation of Pavements Using Embedded Transducers on a Semi-Trailer Suspension

**DOI:** 10.3390/s24237556

**Published:** 2024-11-26

**Authors:** William Levesque, André Bégin-Drolet, Julien Lépine

**Affiliations:** 1 Department of Mechanical Engineering, Laval University, Québec, QC G1V 0A6, Canada; andre.begin-drolet@gmc.ulaval.ca; 2 Department of Operations and Decision Systems, Laval University, Québec, QC G1V 0A6, Canada; julien.lepine@fsa.ulaval.ca

**Keywords:** energy consumption, heavy vehicles, rolling resistance, pavement type, road roughness, road management

## Abstract

Road agency initiatives to reduce traffic-related greenhouse gas emissions are limited by the inability of current experimental methods to assess pavement impacts on vehicle energy consumption. This study addresses this by examining the rolling resistance of a semi-trailer suspension under highway conditions using a precise measurement system with embedded transducers. Data were collected over 174 km of highway, covering various pavement types under mild summer conditions. The analysis revealed notable differences in rolling resistance due to pavement characteristics, with more pronounced variations observed within pavement types than between them. For instance, geographically consecutive jointed rigid pavements showed a 34% variation in rolling resistance, likely correlated with harmonic excitations generated by slab presence, while flexible pavements exhibited up to a 21% variation under similar tire operating conditions. Composite pavements generally performed the worst, possibly due to interactions between bituminous materials and older cement-based foundations. The study also highlighted the critical role of tire operating conditions, showing a decrease of 0.09 kg/tonne in rolling resistance for every 1 °C increase in temperature. This research shows that precisely measuring the rolling resistance (±0.1 kg/tonne) in situ for heavy vehicles is feasible and underscores the need for additional data in diverse weather scenarios to better align laboratory results with on-road realities.

## 1. Introduction

Greenhouse gas (GHG) emissions from heavy vehicles were projected to reach record levels in 2023, with this upward trend expected to continue in the years to come [1,2]. In response, various governmental organizations have undertaken studies to assess how pavement types and characteristics affect fuel consumption in heavy vehicles, with the aim of integrating this knowledge into asset management strategies [3,4,5].

These initiatives were driven by the premise that a small systematic change in the fuel consumption of all heavy vehicles circulating on a pavement can create a significant impact on the scale of the whole heavy-vehicle traffic. Indeed, some authors suggested that the energy consumption during the pavement-use phase (i.e., the fuel consumption of vehicles circulating on it) is orders of magnitude higher than the energy consumption during the pavement-construction phase [6]. For instance, a model-based study simulated the impact of increasing the modulus of elasticity in pavement top layers through resurfacing 10% of the U.S. road network annually, concluding that such efforts could potentially reduce GHG emissions by 0.5% across the entire U.S. transportation sector [7]. This study did not consider other pavement characteristics such as macrotexture and road roughness [8].

Furthermore, traffic distribution and the resulting GHG emissions are highly heterogeneous across road networks [9]. This implies that the necessary improvements to reduce rolling resistance would only need to be implemented for a small portion of the road network in order to achieve a meaningful reduction in GHG emissions from the transportation sector.

**Figure 1:**
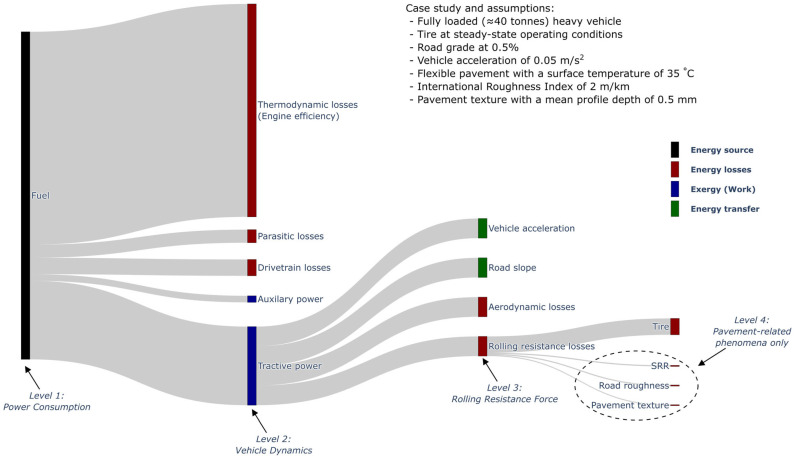
Sankey diagram of the power consumption for a given heavy vehicle and indications of the different levels at which measurements under real driving conditions can occur.

The ambition of making road networks more performant with respect to vehicle GHG emissions has been significantly hindered by the inability of current experimental methods to precisely assess the impact of pavements on vehicle energy consumption [3,4,5,8]. The main reason is the difficulty in isolating rolling resistance from all other factors influencing power consumption, which also depends on the “level” at which the measurements occur. Figure 1 presents a Sankey diagram of power consumption illustrating a case study of a fully loaded heavy vehicle (≈40 tonnes) accelerating uphill on a typical asphalt concrete pavement under summer highway conditions.

Measuring rolling resistance at level 1 using vehicle operational data (e.g., from the engine control unit) results in an indirect measurement method that encompasses all factors influencing power consumption. This includes various factors unrelated to vehicle dynamics, such as thermodynamic losses due to engine inefficiency inherent in internal combustion engines, as well as electrical and mechanical losses (e.g., parasitic losses, drivetrain inefficiencies), and the power consumed by auxiliary systems (e.g., air conditioning).

Measuring rolling resistance at level 2 involves directly measuring tractive power with transducers, producing values that fluctuate due to various aspects of vehicle dynamics, including aerodynamic resistance, potential (i.e., road slope) and kinetic (i.e., vehicle acceleration) energy variations, and external factors such as curves and driver behaviour.

On the other hand, measuring at level 3 means measuring the rolling resistance force where it occurs, which suppresses the need for extra modelling of vehicle power consumption or vehicle dynamics. Level 4 measurements involve assessing the pavement/vehicle interaction and calculating its associated power dissipation. This method has the advantage of being independent of the tire’s contribution to rolling resistance, which is the primary source of the overall rolling resistance [10]. Many experimental studies have been performed to evaluate the rolling resistance of pavements at different levels of measurement with variable success, as presented in Table 1.

The coast-down test method has been used to quantify the rolling resistance of heavy vehicles as a function of pavement macrotexture and international roughness index (IRI) [5]. It was found that the results were “unstable”, and no definitive conclusions could be drawn [5]. Some authors have shown that the coast-down test method has a maximum reproducibility error of 0.6 kg/tonne in the rolling resistance coefficient [28], representing more or less 10% of the total rolling resistance of a heavy vehicle. Given that coast-down tests require a dedicated track, and that the influence of the pavement on rolling resistance is small for individual vehicles [8], this method does not appear suitable for evaluating the rolling resistance of pavements on public roads.

The level 1 method (i.e., vehicle operational data) can be used with either internal combustion engines or electric motors. The analytical frameworks applied to such experimental data for investigating the effect of pavements can be roughly divided into three categories:

- Correlations between power consumption data and some pavement characteristic metrics [3,4,11,12]. This focuses on the relative value of the pavement contribution in relation to a baseline energy consumption without emphasizing the physical interpretation.
- Normalization of power consumption data to isolate the contribution of the pavement using mechanistic models (e.g., aerodynamic resistance, tire rolling resistance, road slope, etc.) and consideration for many parameters such as vehicle speed, wind conditions, and vehicle inefficiencies [17,18]. This focuses on the absolute value of the pavement’s contribution to energy consumption by emphasizing the physical interpretation
- Data mining on power consumption data using more advanced statistical and machine learning methods such as artificial neural networks, support vector regression, and random forest analysis [13,15,16]. This focuses on the statistical significance of the pavement’s contribution to energy consumption rather than emphasizing the physical interpretation.

There is broad agreement in the literature that fuel consumption correlates with the IRI [11,12], although the IRI is not the only road characteristic influencing vehicle fuel consumption [8]. Correlations developed by some authors between fuel consumption and pavement types were found to be significant [3], but other authors found the opposite which suggests that missing variables need to be considered [4] when assessing power consumption [3,5].

Normalization of power consumption has been performed on passenger electric vehicles using an Internet of Things platform [17,18]. Data from 10 nominally identical passenger car vehicles over highways and urban roads was collected, and it was concluded that an increase of 1 m/km in the IRI leads to an increase in “normalized energy consumption” of 3.4% [17].

Data mining on the fuel consumption of 473 heavy vehicles has been performed to develop a neural network of 14 inputs with two hidden layers, which led to a difference in measured and predicted fuel consumption of 1.5% [16]. However, no parametric or sensitivity analysis has been performed on such models, limiting their usefulness for road agencies and pavement engineers.

The main advantage of level 1 methods is that they are relatively simple to deploy because they use existing vehicle operational data. However, one fundamental limitation of level 1 methods for evaluating the rolling resistance of pavements is the fact that the physical quantity of interest (i.e., the contribution of the pavement to power consumption) is diluted by all aspects influencing power consumption. This leads to an uncertainty addition, and it creates a trade-off between the models’ accuracy and their comprehensiveness. For road agencies to justify the necessary investments in potentially reducing rolling resistance attributable to pavements, both accuracy and comprehensiveness are necessary.

Level 2 experimental methods (i.e., driving torque measurements) partially solve the uncertainty addition problem by eliminating the effect of vehicle-related inefficiencies that are independent of vehicle dynamics. Torque measurements at the driving shaft of a heavy vehicle have been used to model the driving losses (e.g., rolling resistance, aerodynamic resistance) under real driving conditions [19], and recent developments have been made to make torque measurements of heavy-vehicle driving wheels more practical [20]. However, driving-torque measurements still require a full model of vehicle dynamics in order to isolate and quantify the rolling resistance [20]. Another challenge specific to driving torque measurements is the trade-off between the sensitivity and fatigue life of the instrumentation. Indeed, if a wheel torque transducer is installed on a driving wheel, the rolling resistance corresponds to a relatively small driving torque of about 0.5 kNm per wheel (for a 40-tonne heavy vehicle with a 5 kg/tonne rolling resistance coefficient, equipped with two driving wheels of 0.5 m radius). However, the instrument must also sustain the braking torque, which can reach up to 35 kNm [20]. While commercial wheel-torque transducers exist and are used in various applications, their effectiveness as devices to precisely determine rolling resistance in steady-state conditions remains to be demonstrated.

The technical challenge of low sensitivity became more apparent during the development of a novel level 3 method. Strain gauges were installed on a heavy vehicle axle in order to measure the axle strains caused by the longitudinal rolling resistance force and the vertical weight [21]. This method appeared to be successful for weight measurements (i.e., vertical direction) but was found to be ineffective for rolling resistance measurements (i.e., longitudinal direction). One possible explanation is the fact that heavy vehicle axles are designed to withstand high stresses, such as cargo loads and braking forces, which are orders of magnitude higher than rolling resistance forces. This means that the rolling resistance caused only a very small strain on the axle, which could make the instrumentation easily spoiled by various sources of noise and errors [29,30]. This is, to a lesser extent, the same challenge with driving-torque measurements.

A successful level 3 method for rolling resistance measurements of passenger vehicle tires under real driving conditions was developed at the Technical University of Gdańsk [31]. It consisted of a dedicated trailer equipped with a passenger car tire mounted on an instrumented vertical arm with a patented counterbalanced system to compensate for road slope and acceleration, all in an encapsulated space. A great deal of literature has been published with data collected from this system [22,23,24,32,33,34], and key insights emerged:

1. The pavement surface can have a significant effect on the rolling resistance coefficient, with differences of about 30% [22].
2. Rolling resistance measurements performed in laboratory conditions, in accordance with ISO and SAE standards, correlate poorly with measurements performed on actual roads [35].
3. Tire operating conditions (e.g., tire temperature and inflation pressure) have a significant effect on the rolling resistance coefficient, and the air temperature limits set by ISO and SAE standards for laboratory measurements are not always representative of real driving conditions [32,36].

This level 3 method can accurately evaluate the rolling resistance performance of pavements but was designed to only provide information for passenger car tires. For this reason, this level 3 method does not yield information on certain phenomena specific to heavy vehicles, such as the structure-induced rolling resistance (SRR) [37]. Moreover, it is believed that the construction of this instrumentation makes it insensitive to road roughness [22].

A level 4 method for heavy vehicles has been developed using a traffic speed deflectometer (TSD), which incorporates Doppler lasers to measure pavement deflection beneath a moving wheel [26]. This method has been used to quantify the SRR coefficient on a 10-tonne axle at two pavement temperatures [27]. The measured SRR coefficient was 0.14 kg/tonne and 0.23 kg/tonne at pavement temperatures of 18 °C and 35 °C, respectively, or around 2.8 and 4.6% of the total rolling resistance (assuming a total of 5 kg/tonne rolling resistance) [27]. Although very precise, the TSD method does not provide a complete understanding of the pavement’s contribution to rolling resistance in heavy vehicles, as it is independent of road roughness and pavement macrotexture.

Hence, there is a need for a precise and direct measurement method for the rolling resistance of heavy vehicles under real driving conditions to accurately evaluate the performance of pavements in that regard. No existing level 3 method simultaneously accounts for SRR, macrotexture, and road roughness all at once to fairly compare in situ pavement type, which has been identified as an important gap in the existing literature.

## 2. Materials and Methods

This paper presents experimental measurements of rolling resistance in a semi-trailer suspension using a novel measurement system. The instrumentation used for this research has been developed in the context of a research project with the Quebec Ministry of Transport and Sustainable Mobility (MTMD). The development of this novel measurement system has been motivated by the need to compare pavement types in terms of rolling resistance under real driving conditions on actual roads. Therefore, the measurements require all tire conditions to be kept the same (e.g., tire temperature, tire pattern, tread wear, aging, etc.).

The measurement system was installed on the central axle of a three-axle flatbed semi-trailer provided by a local training center, the *Centre de Formation en Transport de Charlesbourg* (CTFC), Quebec, QC, Canada. The central axle was chosen because it was expected to undergo smaller slip angles during turns and fewer disturbances, such as aerodynamic drag, due to its proximity to the first axle. The validity of measuring the rolling resistance solely at the central axle of the semi-trailer is appropriate for the scope of this study for the following reasons:

1. All the tires of the heavy vehicle follow the same track (i.e., right and left). This means that they are all exposed to the same macrotexture of the pavement.
2. Road roughness is typically quantified using the IRI, which is based on the simulation of a quarter-car model [38]. This means it is not common practice to consider the whole vehicle when assessing the effect of road roughness.
3. Different wheels of the same heavy vehicle do not interact with each other in terms of SRR [37].

Different coefficients of rolling resistance on the other axles of the heavy vehicle could be obtained if tires of a different model or age were installed. However, such considerations related to tire characteristics are not within the scope of this present study.

**Figure 2:**
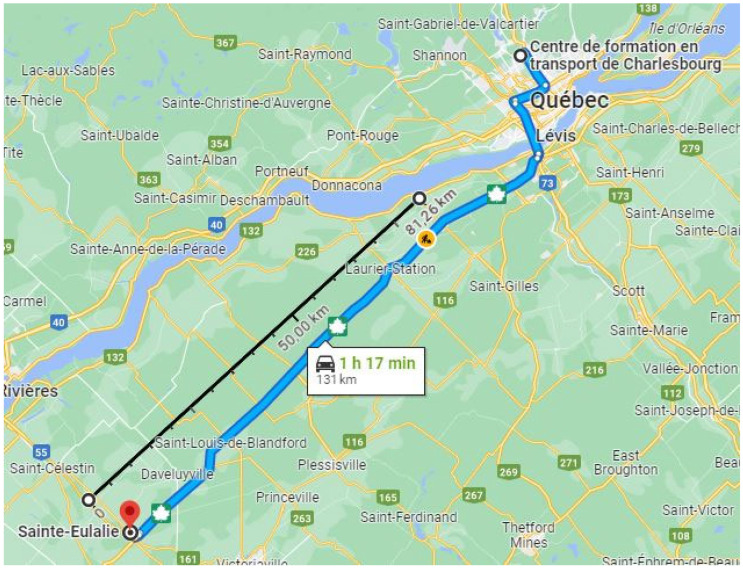
Map of the route travelled by the semi-trailer equipped with the measurement system on 4 October 2023 in Quebec, QC, Canada.

The semi-trailer was equipped with pneumatic suspensions, and the three-axle group was loaded at 18.8 tonnes. The instrumented suspension was equipped with retreaded dual tires of different categories (i.e., steer axle and drive axle tires). The experimental campaign was conducted on 4 October 2023 in Quebec, QC, Canada. The conditions were dry with an air temperature of around 23 °C. Measurements were conducted in highway conditions at a constant speed of 95 km/h. The overall route is presented in Figure 2.

In the route illustrated in Figure 2, there were three categories of pavement types defined in the MTMD’s database. Many results presented in this paper are aggregated in order to evaluate the effect of the three pavement types on the measured rolling resistance.

1. Flexible: pavement with top layers made of bituminous materials on a granular base.
2. Rigid: pavement made of jointed cement concrete slabs on which vehicles roll directly. In the dataset, all rigid pavement surfaces feature longitudinal grooving.
3. Composite: pavement with top layers made of bituminous materials that were installed on an older cement-base foundation.

The specific pavement structure, including layer thicknesses and moduli, can vary within a single pavement type. However, such information was neither consistently recorded nor readily available across Quebec’s road network.

### 2.1. Conceptual Approach of the Measurement System

**Figure 3:**
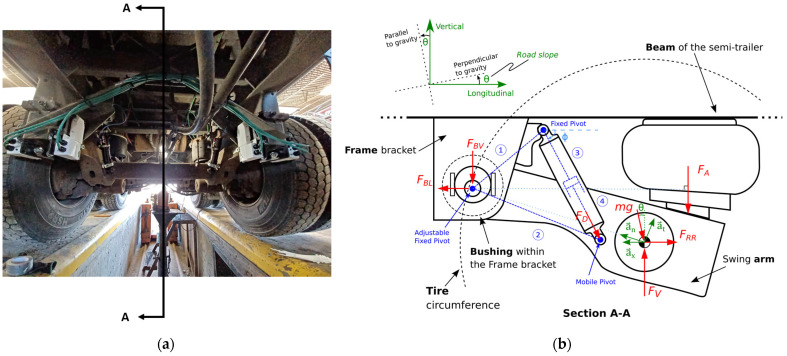
(**a**) Measurement system for every force and displacement between a semi-trailer suspension and its frame used for evaluating the rolling resistance force at the tire/pavement contact patch; (**b**) Free-body diagram of the upward-moving semi-trailer suspension: forces, acceleration vectors, and four-bar mechanism during accelerating uphill travel.

The measurement system was developed to directly measure the rolling resistance force, as defined by level 3 in Figure 1. The measurement system consisted of a variety of transducers that allowed the measurement of every force and displacement between a semi-trailer suspension and its frame. The rolling resistance force was computed by solving the equations of the free-body diagram of the suspension [39]. The measurement system installed on the semi-trailer is presented in Figure 3a along with the underlying conceptual model for rolling resistance computation in Figure 3b.

The whole conceptual approach has been presented in previous work [39]. This paper presents the calculation of the rolling resistance using a straightforward approach, namely by averaging the longitudinal force measured at the frame bracket ($F_{BL}$) and the longitudinal component of the damping force ($F_{D}$) using the average damper angle (ϕ) when the vehicle is at a constant speed. This approach was based on the principle that, over a sufficiently long distance, both the impact of noise induced by road slope and vehicle acceleration on the measured average rolling resistance force, as well as the average road slope itself, would approach zero. This assumes that the cumulative effects of positive and negative road slopes balance out on a road segment with an initial and final point at the same altitude, resulting in a net average road slope of zero. The rolling resistance force ($F_{RR}$) for a half-axle was calculated this way, shown as follows:

(1) $$F_{RR}=avg(F_{BL})+avg(F_{D})\cos\left(\phi \right)$$

In Equation (1), the letters “avg” refer to the average value of the data along the selected road segment or within the current averaging window. The full model illustrated in Figure 3b will be implemented in future work in parallel with the accumulation of data under more varied conditions. This limits the scope of the results presented in this paper to the following hypotheses [39]:

1. The bushing within the frame bracket was considered rigid and represents the fixed pivot point of the suspension motion.
2. The effect of the roll motion of the suspension was considered negligible, which justifies a two-dimensional model.
3. The damper orientation was considered to be aligned with the vertical plane and its angle *ϕ* was assumed constant at 70 deg.
4. The air bellow between the beam of the semi-trailer and the swing arm of the suspension was considered to only transmit a force (*F_A_*) in the vertical direction of the vehicle reference frame.
  - This assumption was based on the uniform inflation pressure within the air bellow and its attachment to the beam of the semi-trailer.
  - Additionally, the flexible material of the air bellow was assumed to transmit no shear forces due to its lower longitudinal stiffness compared to the bushing.

The measurement system (i.e., level 3) presented in Figure 3 has several advantages compared to methods based on vehicle operational data (i.e., level 1) or driving torque measurements (i.e., level 2):

- Insensitive to power unit and power train efficiencies. As discussed in the introduction section, measuring the rolling resistance where it occurs (i.e., using a level 3 method) greatly limits the uncertainty addition as it does not depend on the power unit or the power train (e.g., level 1 method).
- Virtually insensitive to aerodynamic resistance. Since the measured forces are internal, they are not affected by the aerodynamics of the whole semi-trailer as in level 1 or 2 methods, but are influenced only by the aerodynamics of the suspension itself. The measured forces are influenced by the “fan effect” of the wheels rotating in the air [40], which can arguably be considered as part of the rolling resistance. The aerodynamic drag of the axle was assumed to be negligible, especially because the central axle was chosen.
- Low influence of road slope. The force transducers do not perceive the longitudinal gravitational force applied on the whole vehicle due to a non-zero road slope; they only measure the proportion related to the suspension mass. It was assumed that in the case of a non-zero road slope, the longitudinal gravitational force applied on the semi-trailer frame was compensated by the increase in tractive force from the tractor at the kingpin (for a constant vehicle speed). Moreover, the effect of the road slope on the weight supported by the suspension was assumed to be negligible in the context of this study. To illustrate, if a heavy vehicle travels on a positive 0.5% road slope, the longitudinal gravitational force applied on the whole heavy vehicle would be more or less the same as the total rolling resistance force, since a rolling resistance coefficient of 5 kg/tonne is a typical value [28]. This would be the effect on the measured value in a level 1 or level 2 method. However, for a 550 kg suspension [11] using the proposed level 3 method, the longitudinal force perceived by the transducers for a half-axle would be as follows:
  (2) $${\Delta F}_{BL}^{slope}=\frac{550\;[kg]\cdot 9.807[m/s^{2}]}{2}\cdot 0.5\%=13.5\;[N]$$
  The relative significance of this bias error varies with cargo loading but would be a few percent for a loaded axle. More importantly, the bias error on the measured longitudinal force due to road slope should tend to zero if it is averaged over a longer distance with no altitude gain, since the average road slope will tend to zero as well.
- Low influence of vehicle acceleration. The vehicle acceleration perceived by the force transducers is attributable only to the suspension’s inertial resistance. For example, if a heavy vehicle increases from 0 to 100 km/h in 60 s, the vehicle acceleration would be around 0.5 m/s^2^, and the inertial force of the whole heavy vehicle would then be around one order of magnitude higher than the rolling resistance force during this 60s (assuming a 5 kg/tonne rolling resistance coefficient). Again, this would be the effect on the measured value in a level 1 or 2 method. However, for a 550 kg suspension [11] using the proposed level 3 method, the longitudinal force perceived by the transducers for a half-axle would be as follows:
  (3) $${\Delta F}_{BL}^{acceleration}=\frac{550\;[kg]}{2}\cdot 0.5[m/s^{2}]=138\;[N]$$
  This value at high vehicle acceleration is less than the expected rolling resistance force for a loaded half-axle. This worst-case example illustrates the capacity of the measurement system to be relatively insensitive to vehicle acceleration, especially when the speed regulator is used under highway conditions.
- Minimization of mechanical crosstalk. A primary technical challenge in measuring the rolling resistance under real driving conditions is the significant disparity between the vertical and longitudinal forces acting on a wheel, with the vertical force being approximately 200 times greater than the longitudinal force (assuming 5 kg/tonne of rolling resistance). This ratio, referred to as the force ratio, presents a challenge for measurements. If strain gauges are installed on a wheel and an encoder is used to estimate the longitudinal force, the resulting transducer must accurately measure and isolate a force that constitutes only 0.5% of its total load capacity, assuming the cargo load is significant enough to match the capacity of the wheel rim material. This is problematic because some commercial load cells typically exhibit a combined error (due to nonlinearity, hysteresis, repeatability, and creep) of about ±0.05% of their capacity, which represents approximately 10% of the force that needs to be measured in this case. The measurement system developed in this study effectively minimized mechanical crosstalk by leveraging two key factors: the air bellow’s inability to transmit shear forces and the placement of force transducers farther from the axle at the frame bracket rather than on the wheels or axle. This increased distance reduced the force ratio from approximately 200 to 10. Additionally, the force transducers installed at the frame bracket did not need to be axisymmetric (as is required for a wheel), allowing for higher sensitivity (and lower strength) in the longitudinal direction compared to the vertical direction, as presented in a previous study [39].

The minimal influence of the road slope and vehicle acceleration using the measurement system was expected to reduce the distance over which the measured values needed to be averaged. Consequently, the resolution in evaluating the rolling resistance of pavements was expected to improve compared to level 1 methods (e.g., vehicle operational data) and level 2 methods (e.g., driving torque measurements).

### 2.2. Force Measurements at the Frame Bracket

**Figure 4:**
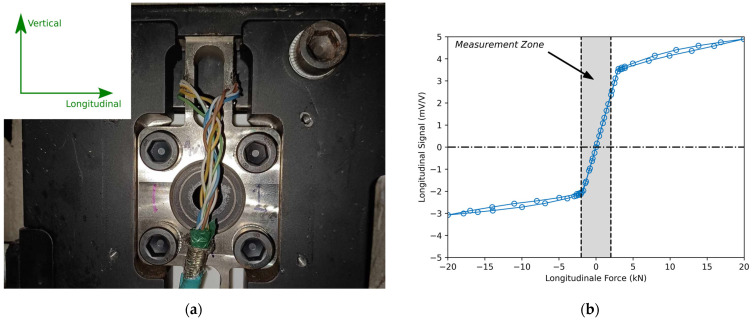
(**a**) Custom titanium load cell installed on the semi-trailer suspension to measure two orthogonal forces with an embedded mechanical end stop on each side; (**b**) Calibration and braking-force test of the custom titanium load cell in a laboratory using an electric traction machine. Measurement zone and zero signal value indicated by dotted lines and dash dot line respectively.

The centrepiece of the measurement system is a custom titanium load cell installed on each side of each frame bracket. The alloy of the load cells was Ti-6AL-4V. This load cell was designed to measure two orthogonal forces (i.e., longitudinal and vertical) and sustain braking forces without failure by using an embedded mechanical end stop. The latter is of ultimate importance to ensure the safe use of the system on actual roads. The concept was tested using an MTS Insight electric traction machine with a 100 kN capacity. This experience consisted of applying a variable longitudinal force of ±20 kN on the load cell and observing the evolution of the measurement signal. These laboratory results and the installation of the custom titanium load cell are presented in Figure 4.

Figure 4a presents the assembly of the custom load cell installed on each side of the frame brackets of the semi-trailer suspension. The central region with a higher slope in Figure 4b corresponds to the measuring region of the load cell from which the results presented in this paper originate. The transition from a higher slope to a lower slope corresponds to the point of application in which the load cell has enough longitudinal displacement to be directly in contact with the surrounding parts of its assembly, hence being in contact with the mechanical end stop. This could happen in practice in the context of braking or from a punctual impact on the suspension (e.g., hole). The slight asymmetry seen in Figure 4b is explained by the uncertainty of the fabrication process. From this laboratory experiment, it can be concluded that the custom load cell presented a capacity in the longitudinal direction of ±2 kN with a sensitivity of approximately 2 mV/V, and could sustain forces beyond ±20 kN.

### 2.3. Asymmetric Damping Effect

Another important aspect of the measurement system is related to the displacement and the force occurring in the dampers of the moving suspension. Indeed, road roughness is the cause of suspension displacements, which dissipates energy, notably in the dampers. Based on current literature [8], it was hypothesized that this would increase the vehicle’s energy consumption. This was considered by measuring both the damper displacement and the resulting damping force (*F_D_*).

Some theoretical studies have investigated the relationship between suspension power dissipation and vehicle characteristics using a quarter-car model and white noise to represent the road profile [41]. It was demonstrated that power dissipation within the suspension is proportional to tire stiffness and noise intensity (i.e., road roughness), but independent of all masses and suspension parameters [42]. On that premise, the choice of a specific damper model for the measurements was not considered significant.

The damper displacement was measured using a linear potentiometer installed on the damper itself [43]. This was believed to be a simple and robust way to assess the effect of road roughness using in-vehicle sensors, compared to other methods, such as accelerometers installed on the vehicle chassis [44].

**Figure 5:**
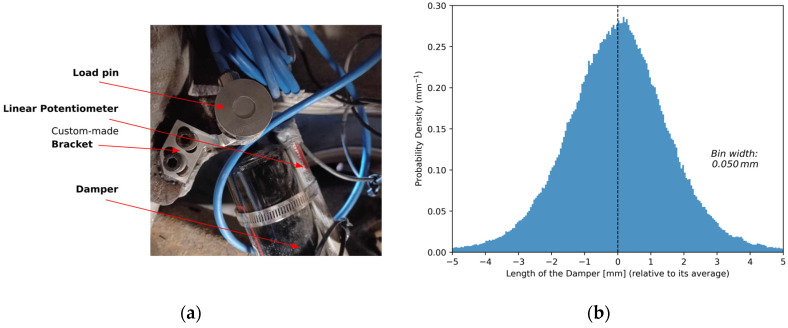

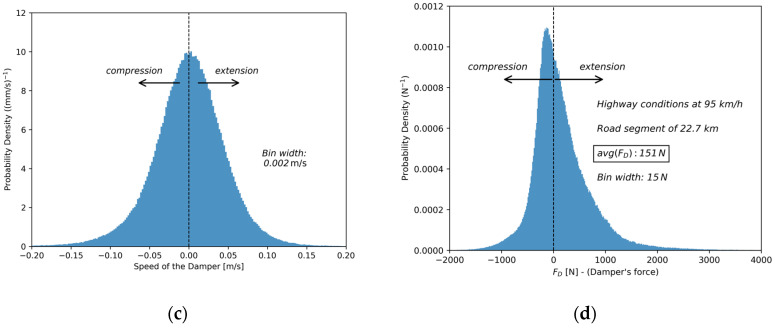
(**a**) Picture of the load pin that measures the damping force and the linear potentiometer that measures the damper displacement; (**b**) Probability density function of the deviation from the average damper length; (**c**) Probability density function of the damper speed; (**d**) Probability density function of the damping force measured by the load pin.

The damping force in the damper (*F_D_*) was measured using a commercially available load pin that was custom-built to replace the pivot bolt [45]. The load pins were constrained in rotation and axially with an aluminum bracket designed for this purpose, which is illustrated in Figure 5a.

The damping force (*F_D_*) in Equation (1) is defined as positive when the damper is in extension, and negative when in compression. This sign convention was chosen because the damper is not vertically oriented; when working in extension, it actively pulls the vehicle against its displacement and, inversely, pushes it forward when in compression. Moreover, the damper in the measurement system has an asymmetric damping coefficient (i.e., higher in extension than compression), which can be summarized as follows:

(4) $$\begin{array}{c} {{(F}_{D})}_{extension}=C_{extension\;}[N/\left(m/s)\right]\cdot \frac{dx}{dt}\;[m/s]\\ {{(F}_{D})}_{compression}=C_{compression}\;[N/\left(m/s)\right]\cdot \frac{dx}{dt}\;[m/s] \end{array}$$

where $C_{extension}>C_{compression}$, which are the two values of the damping coefficient, and $\frac{dx}{dt}$ is the derivative of the damper length with respect to time. To the best of the authors’ knowledge, this difference in damping coefficient is typical of semi-trailers’ dampers.

Based on Equation (4), it was not assumed that the average value of $F_{D}$ would tend to zero over a given period of time. The average damping force $F_{D}$ could differ from zero because the frame bracket, from which the forces $F_{BL}$ and $F_{BV}$ originate, acts more like a rigid pivot than a linear spring. Hence, it is possible that there is an interaction between the forces at the frame bracket ($F_{BL}$, $F_{BV}$), the force at the air bellow ($F_{A}$), and the damping force ($F_{D}$), ensuring that the condition of no average acceleration between the suspension centre of mass and the semi-trailer frame is respected.

This idea can be better visualized via the probability density function (PDF) of the measured load pin force and damper displacement. Indeed, the damper displacement and speed PDF were expected to follow a centred normal function, since the average acceleration of the suspension relative to the semi-trailer frame must be zero. From this, an asymmetric damping coefficient, as indicated in Equation (4), applied to the damper speed PDF should result in an asymmetric PDF of the damping force. This is exemplified in Figure 5 using experimental data collected over a 22.7 km road segment.

The PDF in Figure 5d, obtained experimentally, appears different from what theoretical studies using mass-damper-spring models and a constant damping coefficient seem to suggest [46]. Vehicle dynamics, and more specifically, suspension dynamics, are typically modelled as a combination of vertical translation movements and oscillations of the vehicle chassis [47,48]. The free-body diagram in Figure 3b and the experimental data in Figure 5 suggest that a semi-trailer suspension could be more accurately represented as a pendulum, with the forces at the pivot (*F_BL_*, *F_BV_*) varying according to the suspension motion. This means that the forces between the suspension and the semi-trailer frame vary over time in at least two orthogonal directions: longitudinal and vertical. However, further modelling of this concept lies beyond the scope of this study.

Nonetheless, the fact that the average damping force is not zero is a key aspect of this research, because the calculation of the rolling resistance relies on the measurement of all forces between the suspension and the semi-trailer frame, as indicated in Figure 3b. The contribution of the non-zero average value of *F_D_*, in the longitudinal direction, is called the “Longitudinal Asymmetric Damping Effect” throughout the paper and is the product of $avg\left(F_{D}\right)\cos\left(\phi \right)$. This corresponds to the second term of Equation (1), which allows for the calculation of the rolling resistance at the tire/pavement contact patch. The inclusion of the Longitudinal Asymmetric Damping Effect enables reliable rolling resistance measurements, even when road roughness significantly excites the suspension, which is often the case on public road networks.

### 2.4. Tire Operating Conditions

**Figure 6:**
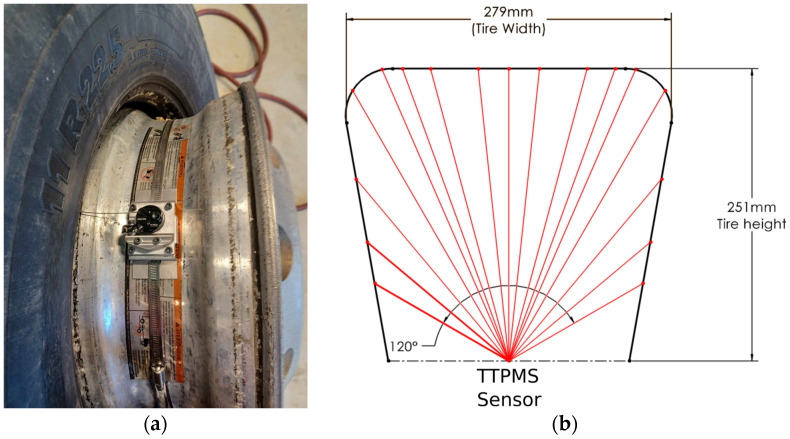
(**a**) Truck tire temperature and pressure monitoring system installed at the centre of the rim; (**b**) Conceptual representation of the 16 channels (red lines) used to monitor the tire’s internal temperature.

The existing literature showed that tire operating conditions such as internal temperature and inflation pressure can significantly affect rolling resistance [10,49]. This is an important aspect since a measured variation of rolling resistance due to the evolution of tire operating conditions should not be attributed falsely to the effect of pavement characteristics. The significance of the tire in the measured rolling resistance is visually represented in the Sankey diagram of power consumption shown in Figure 1. To account for these effects, a truck tire temperature and pressure monitoring system [50] was installed on the wheel rim on each side of the suspension. The TTPMS measured, at 0.1Hz, the inflation pressure and the internal temperature of the tire at 16 different points along its transversal section. Measuring the tire temperature at different points can be useful since it is not uniform [32,51], and some authors found that the temperature at the sidewalls correlates better with rolling resistance than the temperature near the apex [52]. The TTPMS allowed the comparison of selected road segments in terms of tire temperature and facilitated the warm-up period at the start of the measurements. Figure 6 illustrates the TTPMS mounted on the rim of the wheel prior to replacing the tire over the rim. The label on the rim visible in Figure 6 was already present and is not pertinent for the current study. As per standard practices by the CFTC, the wheels of the semi-trailer were not balanced after the installation of the TTPMS.

### 2.5. Data Acquisition and Processing

The data obtained using the measurement system was preprocessed in order to relate pavement types and rolling resistance. The following steps were applied to the dataset collected on 4 October 2023, from Highway 20 in Quebec, QC, Canada:

1. Identification of the road segments with constant vehicle speed (i.e., 95 km/h);
2. Subdivision of the road segments based on pavement types;
3. Verification of the evolution of average tire temperature and road slope.

The first step was to define the road segments where the vehicle speed was constant (i.e., 95 km/h). This was accomplished using the data at 1 Hz from a standalone GPS module [53] installed in the data acquisition system. The usage of the cruise control greatly minimized the forces transmitted between the suspension and the semi-trailer frame due to vehicle acceleration (${\overset{⃑}{a}}_{x}$) [39]. The constant speed segments were obtained using an algorithm in which the bandwidth speed value was 95 ± 5 km/h, and the minimum road segment length was 2.5 km. This means that the cruising speed of the CFTC tractor was set to 95 km/h, and all road segments used for calculating average rolling resistance values were longer than 2.5 km.

The second step was to subdivide every constant-speed segment based on pavement type categories. This was accomplished using the georeferenced database of the MTMD. Thus, a list of 16 constant speed segments with various pavement types was obtained, which is summarized in Table 2.

The third step was to compute the average tire temperature for each segment, along with the average road slope (*θ*) estimated using GPS data. In the dataset of the road segments identified in Table 2, no specific location within the tire for temperature measurements appears more optimal in relation to rolling resistance. For this reason, the internal temperature was obtained by averaging the 16 temperature values provided by the TTPMS sensor, which are illustrated in Figure 6b.

The road slope (*θ*) was calculated to verify that the average for each segment was close to zero. As explained in Section 2.1, an average road slope that tends to zero ensures that the gravitational effects tend to zero as well.

All the force and displacement measurements presented in the previous section were performed using a custom-built data acquisition system. All signals were sampled at 16 kHz and downsampled using uniform averaging to 1 kHz before being saved on an SD card. A low-pass order-8 Butterworth filter with a cut-off frequency of 4 Hz and a mobile averaging window of 10 s was applied to the signals of the load cells installed at the frame brackets. This cut-off frequency was selected as it is approximately half of the wheel rotation frequency, and filtering out this rotation was beneficial because it did not contribute to the average value of the load cell measurements.

For the load pins that measured the damping force, a low-pass order-8 Butterworth filter with a cut-off frequency of 20 Hz, and an averaging window of 10 s was applied to the signals. For the linear potentiometers that measured the damper length, no digital filtering was performed during data processing.

## 3. Results and Discussion

### 3.1. Measured Rolling Resistance

**Figure 7:**
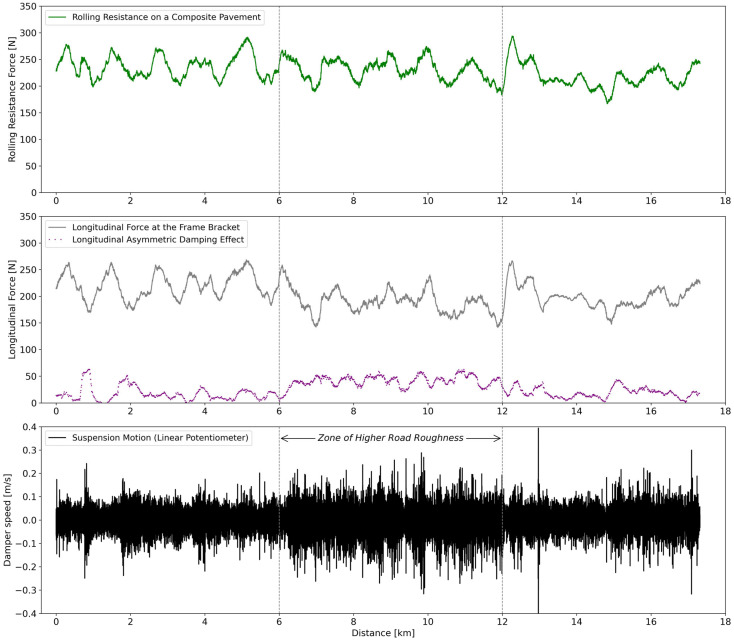
(**Green line**) Calculated half-axle rolling resistance using the speed regulator at 95 km/h on a composite pavement; (**Gray line**) Measured longitudinal force at the frame bracket (*F_BL_*) using two custom titanium load cells installed on a frame bracket; (**Purple dotted line**) Longitudinal Asymmetric Damping Effect $\left[F_{D}\cos\left(\phi \right)\right]$ using a load pin; (**Black line**) Measured damper speed using a linear potentiometer.

The longitudinal force (*F_BL_*) measured at the frame bracket is the most important value for estimating rolling resistance with the measurement system. Indeed, a semi-trailer suspension at a perfectly constant speed on a perfectly flat road with a roughness of 0 m/km would have a longitudinal force at its frame bracket that is theoretically equal to the rolling resistance force at the tire/pavement contact patch [39]. Another important aspect is the damping force (*F_D_*) in the longitudinal direction. This value was expected to be close to zero most of the time and to increase on the road segments with a higher road roughness. These concepts are exemplified in Figure 7 using data collected over a 17.6 km road segment of composite pavement.

The road slope, which is small but never zero, along with its interaction with the speed regulator, likely causes the variations in the green line (i.e., the rolling resistance force averaged over a moving window of 10 s) seen in Figure 7. Moreover, the interaction between the grey line (i.e., *F_BL_*) and the purple line (i.e., *F_D_*) can be visualized in Figure 7, especially between the distances of 6km and 12km. Indeed, this is a road segment with a higher road roughness which increases the motion of the suspension and the Longitudinal Asymmetric Damping Effect, leading to a decrease in the longitudinal force at the frame bracket (*F_BL_*). The addition of these two forces, as defined by Equation (1), results in a stable and accurate rolling resistance signal, even in the zone of higher road roughness illustrated in Figure 7.

**Figure 8:**
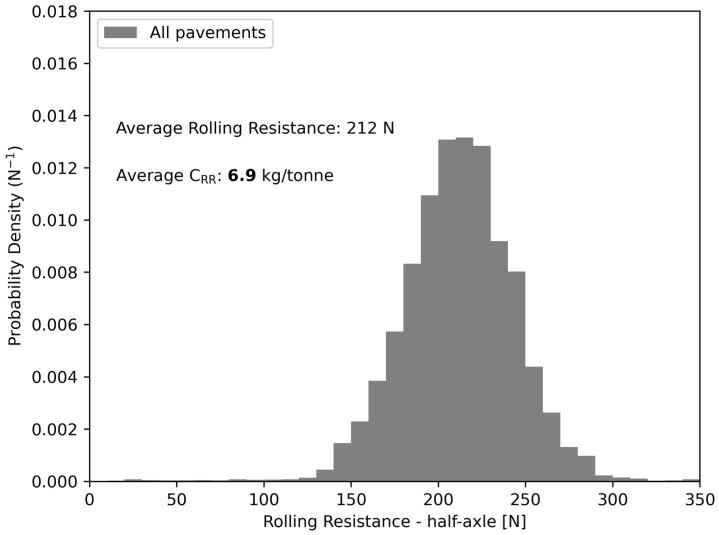
PDF of the measured rolling resistance force of a half-axle on every road segment (i.e., 174.4 km) at a constant vehicle speed of 95 km/h.

The measured rolling resistance, such as the green line in Figure 7, can be averaged over a sufficiently long road segment (i.e., at least 2.5 km) so that the effects of road slope and speed variations virtually tend to zero. An initial demonstration of this concept is presented in Figure 8, where the whole dataset (i.e., 174.4 km long) obtained from the data processing of Section 2.5 was used to build the PDF of the measured rolling resistance force.

Since the obtained PDF is reasonably normal, the average coefficient of rolling resistance can be calculated directly:

(5) $$C_{RR}^{avg}=\frac{212\;[N]}{9.807[m/s^{2}]}\cdot \frac{6\;[\text{half-axle}/\text{three-axle group}]}{18.81\;[\text{tonne}/\text{three-axle group}]}=6.9\;[kg/tonne]$$

Equation (5) makes the implicit assumption that the pneumatic suspension of the three-axle group (i.e., six half-axles) has a perfectly equal weight distribution among its axles. This is a reasonable assumption because the air bellows are connected together and share the same average inflation pressure. The calculated value of the rolling resistance coefficient was within the expected order of magnitude, based on previous literature [49,54].

### 3.2. Uncertainty in the Measured Rolling Resistance Coefficient

Like every measurement system, many sources of errors must be considered in order to define the uncertainty in the measured rolling resistance. Table 3 shows all the potential sources of errors that have been identified, as well as their origin and the resulting uncertainty impact on the rolling resistance coefficient. The discussion in this section explains every source of error identified in Table 3 and concludes with the overall uncertainty.

There is always a resolution when performing an analog-to-digital conversion (ADC). In the context of this study, the measurement zone identified in Figure 4b is ±2000 N/load cell. During the on-road tests, the gain of the signal amplifiers was adjusted to ensure the measurement zone corresponds to the full-scale range of the ADC. Knowing that the ADC is 12 bits and that there are two load cells per half axle, the increment of the measured longitudinal force is calculated as follows:

(6) $$\frac{4000\left[N/load\;cell\right]}{2^{12}-1\left[bit\right]}\cdot 2\;[load\;cell]=2.0\left[N/bit\right]$$

Similarly to Equation (5), the result of Equation (6) can be normalized as a resolution in the measured coefficient of rolling resistance:

(7) $$\frac{2.0[N/bit]}{9.807[m/s^{2}]}\cdot \frac{6\;[\text{half-axle}/\text{three-axle group}]}{18.81\;[tonne/\text{three-axle group}]}=0.065\;\left[\frac{kg/tonne}{bit}\right]$$

Knowing that the bias error of the ADC is half its resolution, this source of bias error was calculated as follows:

(8) $$\varepsilon_{1}=0.065\;\left[\frac{kg/tonne}{bit}\right]\cdot 0.5\;\left[bit\right]=\pm 0.033\;\left[kg/tonne\right]$$

Every data acquisition system can be subjected to random noise caused by various electronic components, which introduces a random error with a zero average. In the context of this study, the rolling resistance signal was averaged over a mobile window of 10 s, and the rolling resistance values for various road segments were obtained over at least 2.5 km. In addition, as explained in Section 2.5, the acquisition frequency was 16 kHz with averaging performed in random-access memory to downsample and save data at 1kHz. This approach prevents the potential effect of spectral aliasing from very high, non-physical frequencies. For these reasons, the effect of random noise was considered negligible:

(9) $$\varepsilon_{2}≅0$$

When the temperature of a load cell changes, a change in signal intensity can occur due to the difference in the coefficient of thermal expansion between the strain gauges and the spring element (i.e., Ti-6AL-4V) [55]. This effect was greatly minimized by the fact that the titanium load cells at the frame bracket were composed of full Wheatstone bridges. For this reason, and considering that the daily air temperature variation was limited, this effect was considered negligible:

(10) $$\varepsilon_{3}≅0$$

The strain gauge manufacturer specifies that the change in the gauge factor as a function of temperature is +1.5%/100 °C. Assuming a cautiously overestimated daily air temperature variation of ±5 °C, the effect of gauge factor variation on the measured rolling resistance was calculated as follows:

(11) $$\varepsilon_{4}=1.5\%\cdot \frac{\pm 5\left[{}^{\circ}C\right]}{100\left[{}^{\circ}C\right]}\cdot 6.9\;[kg/tonne]=\pm 0.0052\;[kg/tonne]$$

Another source of error arises with load cells when the air temperature changes, causing variation in the elastic modulus of the spring element (i.e., Ti-6AL-4V). Since no compensation resistors or other correction methods were employed for this aspect, the rate of change in the modulus of elasticity as a function of temperature for Ti-6AL-4V is 0.05%/°C [56]. Assuming again a daily air temperature variation of ±5 °C, the resulting error in the measured rolling resistance was calculated as follows (the rate of change in the modulus of elasticity as a function of temperature has been expressed as an absolute value in Equation (12), because a reduction in the modulus of elasticity of the spring element leads to an increase in the signal intensity):

(12) $$\varepsilon_{5}=\pm 5\left[{}^{\circ}C\right]\cdot \left|-0.05\right|[\%/{}^{\circ}C]\cdot 6.9\;[kg/tonne]=\pm 0.017\;[kg/tonne]$$

On datasheets of commercial load cells, several types of errors are presented [57]:

- Nonlinearity (ε_6_) is the maximum deviation of the calibration curve from a straight line drawn between the minimum load and 75% of the rated capacity. This is caused by the change in geometry of the spring element under a load.
- Hysteresis (ε_7_) is defined as the maximum amplitude difference in a signal between the ascending and descending curve when measuring a load.
- Nonrepeatability (ε_8_) indicates the maximum difference between load cell signals at repeated loads under identical loading and environmental conditions. This is a random and uncorrelated error.
- Creep (ε_9_) refers to the change in load cell signal occurring with time under constant load and otherwise constant variables.

The four errors mentioned above are relevant to static loading conditions. In the context of on-road measurements, the measured forces varied dynamically across many frequencies, and the quantity of interest was not a specific point, but the mobile average, or the integral of all the points in the measurement zone defined in Figure 4b. For this reason, the four errors mentioned above were considered negligible:

(13) $$\varepsilon_{6}≅0$$

(14) $$\varepsilon_{7}≅0$$

(15) $$\varepsilon_{8}≅0$$

(16) $$\varepsilon_{9}≅0$$

To conservatively combine the errors listed in Table 3, the worst-case method is used, which simply consists of summing up all the errors:

(17) $$\varepsilon =\sum_{i=1}^{9}\varepsilon_{i}=\pm 0.055\;[kg/tonne]$$

In the context of introducing measurement results using a novel and complex system, the uncertainty was rounded upwards to yield only one significant number and one decimal place. This yields the following uncertainty value:

(18) $$Ε={\left\lceil \varepsilon \right\rceil }_{0.1}={\left\lceil \pm 0.055\;\left[kg/tonne\right]\right\rceil }_{0.1}=\pm 0.1\;\left[kg/tonne\right]$$

The uncertainty value calculated in Equation (18) applies to all rolling resistance results presented in this paper. Despite being a prudent estimate of the system uncertainty, this value can be considered very precise compared to other studies using the level 1 method [5,58]. In addition, the European standard for labelling the rolling resistance of tires defines categories within a range of ±$0.5\left[kg/tonne\right]$. This means that the measurement system is precise enough to distinguish between different tire labels under real driving conditions.

The information in this section indicates that the potential error in evaluating the rolling resistance of pavements arises not from the inherent uncertainty of the embedded transducers of the measurement system, but rather from other disturbing factors such as road slope, vehicle acceleration, and tire operating conditions, if they are not considered appropriately.

### 3.3. Pavement Type and Statistical Analysis

**Figure 9:**
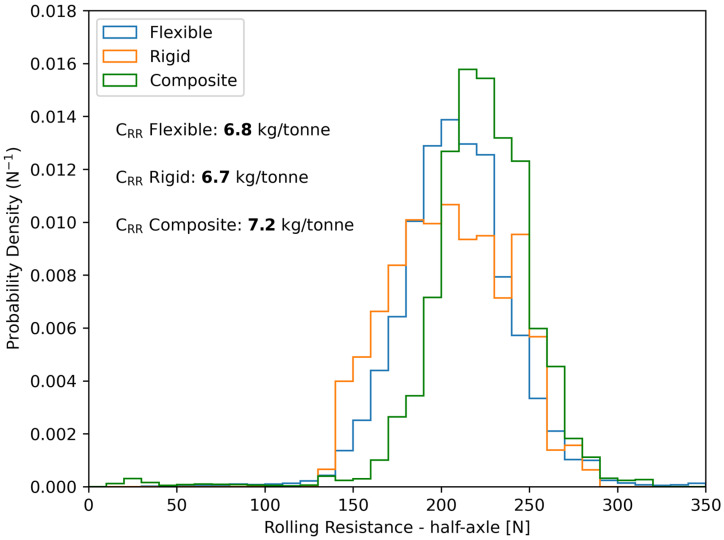
PDFs of the measured rolling resistance of a half-axle on each pavement type at a constant vehicle speed of 95 km/h.

To consider the effect of pavement type on rolling resistance, the PDF in Figure 8 can be divided into three different PDFs, one per category of pavement type. Then, the average force for each PDF can be calculated to see if there is any apparent difference in rolling resistance due to pavement type. This is illustrated in Figure 9.

**Figure 10:**
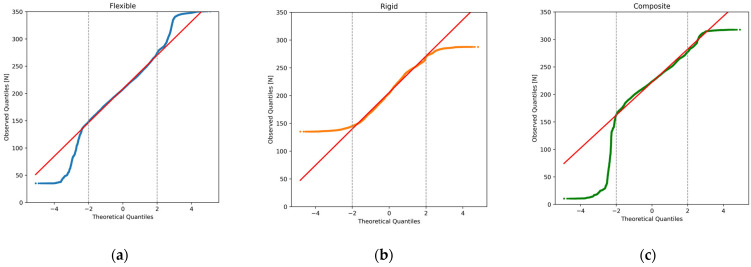
Comparative analysis of the normality of the measured longitudinal force for the three pavement types: Q-Q Plots for (**a**) Flexible, (**b**) Rigid, and (**c**) Composite pavements.

It can be seen in Figure 9 that the average rolling resistance force for the composite type appears to be higher compared to that of flexible and rigid pavement types. Moreover, the rigid type seems to have a higher data spread (i.e., higher standard deviation), which indicates that an additional aspect or phenomenon should be considered for rigid pavements. To further analyze the statistical characteristics of the measured longitudinal force, Q-Q plots were performed for each pavement type, which are presented in Figure 10.

It can be seen in Figure 10 that all distributions follow the redline of normality relatively well in the range of ±2 standard deviations, especially for flexible and rigid pavements. Several phenomena that might not have been fully addressed by the constant speed segmentation algorithm could explain the presence of extreme values in the rolling resistance dataset:

- Coasting events (extremely low values)
- Braking events (extremely high values)
- Vehicle turning (both extreme ends)

Considering that normality is maintained within ±2 standard deviations (i.e., 95% of the data) and the chosen course shown in Figure 2 is straight, the dataset is suitable for further analysis of rolling resistance.

### 3.4. Rolling Resistance Measurements and Disturbing Factors

**Figure 11:**
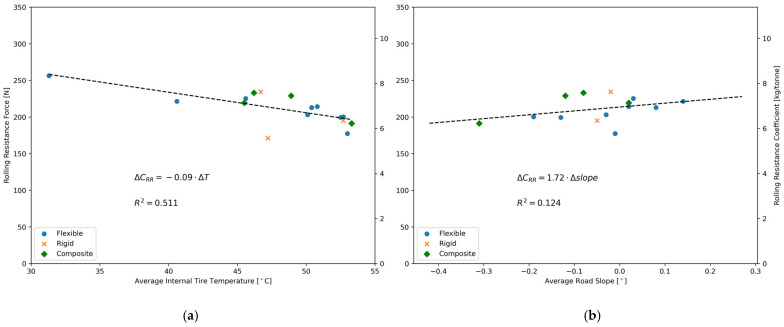
(**a**) Relationship between the rolling resistance of various road segments for a half-axle and the average internal tire temperature; (**b**) Relationship between the rolling resistance of various road segments for a half-axle and the average road slope.

Heavy-vehicle tires require a certain warm-up period during rolling resistance measurements so their internal temperature reaches steady-state conditions [59]. This was considered with the distance travelled between the starting point and Highway 20. Initially, the internal tire temperature was equal to the air temperature (i.e., 23 °C) and reached 31 °C after 33 min at the beginning of the first road segment identified during data processing (see Section 2.5). The maximum measured temperature was 54 °C. From previous literature [49,59], it was expected that an increase in tire temperature would result in a decrease in the rolling resistance coefficient. A direct correlation with tire temperature was defined by computing the average rolling resistance force on the 16 road segments of Table 2 along with their respective average internal tire temperature. This correlation is illustrated in Figure 11.

From Figure 11a, it can be seen that in the range of 31 °C to 54 °C of tire temperature, the C_RR_ decreases by 0.09 kg/tonne for every °C increase in tire temperature. This experimental value obtained using the measurement system was compared with similar correlations found in the literature. Some authors, through laboratory measurements of rolling resistance conducted using a heavy-vehicle tire on a steel drum in a wind tunnel, found a correlation between rolling resistance and tire temperature, which is nearly identical to the one presented in Figure 11a for the tested temperature range [49].

It must be specified that not all road segments in Figure 11 have the same length, which means they do not have the same weight in the probability density functions in Figure 8 and Figure 9. Moreover, from Figure 11a, it can also be seen that the linear correlation is significant, with a coefficient of determination of 0.512. This means that approximately half of the variance in the measured rolling resistance force is explained by other factors, which are believed to be related to pavement characteristics. This is particularly true for rigid pavements.

Figure 11b also shows the relationship between the average road slope of various road segments and their measured rolling resistance. Despite having a lower R^2^ value of 0.124, the increase in measurements by 1.72 kg/tonne per degree of road slope is noteworthy. Indeed, applying the logic from Equation (2) in Section 2.1, an increase in the measured longitudinal force of 47.1 N for a road slope of 1° can be predicted. Using Equation (5) in Section 3.1, this value can be normalized in terms of an equivalent rolling resistance coefficient, which yields a value of 1.53 kg/tonne per degree of road slope. The latter value can be considered an approximate reference as the exact weight of the suspension, set as 550 kg in Section 2.1 based on the literature, is only approximate. The experimental data presented in Figure 11b supports the physical interpretation explained in Section 2.1 regarding the effect of the road slope on the measurements. Finally, it is worth mentioning that two road segments whose combined lengths represented only 4% of the whole dataset were not included in Figure 11b, because they were outliers due to their lower tire temperature, which exaggerated the influence on the linear regression.

Tire temperature and road slope can be considered for each pavement type in order to evaluate their potential importance in the rolling resistance results presented in Figure 9. These two aspects are considered disturbing factors because they can influence the measured rolling resistance, and they are not fundamentally related to pavement characteristics. Following this definition, road roughness is not a disturbing factor.

In ideal conditions, the tire temperature would be perfectly constant and the road slope would be zero. The effect of a tire temperature that deviates from the average value across the 16 road segments (i.e., 48.3 °C) can be compensated using the coefficient of −0.09 kg/tonne/°C presented in Figure 11a. The effect of a non-zero road slope on the measured rolling resistance can be estimated by considering the coefficient of 1.72 kg/tonne/° from Figure 11b. This is summarized in Table 4, with average values for each pavement type.

It can be observed in Table 4 that the combined effect of tire temperature and road slope for each pavement type was either within the uncertainty of the measurement system (i.e., ±0.1 kg/tonne), or significantly smaller. For this reason, no compensation for these disturbing factors was applied to the measured rolling resistance on any of the 16 road segments in the dataset. This decision also facilitates comparisons of rolling resistance between geographically consecutive road segments, reflecting the conditions at the specific time of measurement.

### 3.5. Rolling Resistance and Road Roughness

The suspension motion was expected to be correlated positively with rolling resistance. In order to compare suspension motion and rolling resistance, a metric of the former had to be defined. One possible metric was the standard deviation of the damper speed, which can be calculated from its central normal distribution, such as the one illustrated in Figure 5c). This metric was chosen due to its conceptual similarity to the international roughness index (IRI) [38,60]. One key difference is that, in this study, damper speed was measured directly rather than simulated. Another metric considered was the Longitudinal Asymmetric Damping Effect, defined by the second term of Equation (1) and illustrated in Figure 5d.

**Figure 12:**
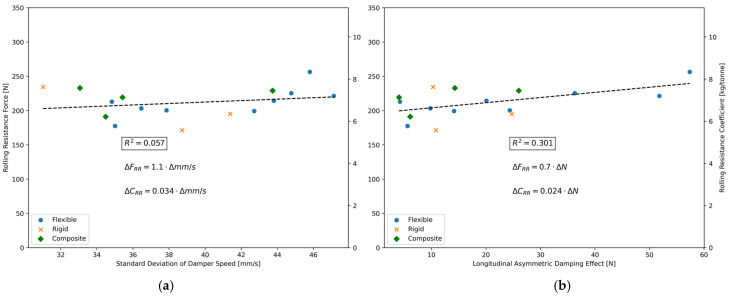
(**a**) Linear correlation between rolling resistance of various road segments and the standard deviation of damper speed; (**b**) Linear correlation between rolling resistance of various road segments and the Longitudinal Asymmetric Damping Effect.

Direct correlations between the average rolling resistance force of the 16 road segments of Table 2 and the two aforementioned metrics were computed and are illustrated in Figure 12.

As shown in Figure 12, rolling resistance demonstrates a positive correlation with suspension motion, which aligns with expectations. A notable observation is the significantly higher R^2^ value (i.e., 0.301) obtained using the Longitudinal Asymmetric Damping Effect compared to the standard deviation of damper speed, which produced a much lower R^2^ value of 0.057, indicating a lower correlation.

This suggests that not all road roughness wavelengths or characteristics—such as fissures, holes, or other irregularities that interact dynamically with suspensions and contribute to the calculation of the international roughness index (IRI)—are directly linked to rolling resistance.

Figure 12 points to the possibility that only the road surface irregularities which cause deviations in the mean damping force are correlated with rolling resistance. Identifying specific road roughness characteristics is beyond the scope of this paper. Further data collection and more comprehensive analyses would be required to address this aspect.

### 3.6. Road Segment Comparison

The average values of tire temperature obtained from the TTPMS sensor, road slope (calculated with GPS data), longitudinal force at the frame bracket [$avg(F_{BL})$], Longitudinal Asymmetric Damping Effect [$avg(F_{D})\cos\left(\phi \right)$], and rolling resistance were computed for each of the 16 road segments presented in Table 2. This information from the whole dataset is summarized in Table 5.

As presented in Table 2, the rolling resistance measured for flexible pavements has a wider range of values (i.e., from 5.8 kg/tonne to 8.3 kg/tonne) than the other two pavement types. This is partly due to the difference in tire temperature correlating with extreme values of rolling resistance. Indeed, the first road segment was before reaching Highway 20, and the tire temperature was still far below its steady-state value. A better comparison would be the road segments 13 and 9, which have rolling resistance coefficients of 5.8 and 7.0 kg/tonne, respectively. This difference of 21% in rolling resistance exists even with a very similar tire temperature between the two road segments. This observation highlights the intra-variability of rolling resistance within the same pavement type, suggesting that factors other than the pavement type can explain the difference in rolling resistance. A more detailed analysis that includes other factors (e.g., pavement age, road roughness, layers thickness, and macrotexture) should be performed in future work to provide more precise insights.

The composite pavements also had a broad range of rolling resistance (i.e., from 6.2 kg/tonne to 7.6 kg/tonne in a relatively constant tire temperature. In addition, three out of four composite pavements along the tested course exhibited a high rolling resistance (i.e., 7.1, 7.3, and 7.5 kg/tonne). Considering that flexible pavements had a smaller average rolling resistance of 6.7 kg/tonne, it suggests that the characteristics of the pavement structure can have a significant impact on the rolling resistance of heavy vehicles even though it is not directly in contact with the vehicle wheels. This is consistent with theoretical studies on SRR modelling [21,61].

The rigid pavement category presented a low rolling resistance for two road segments (i.e.,5.6 and 6.4 kg/tonne) out of three. This suggests that rigid pavements have the potential to be a performant choice in terms of rolling resistance. However, one road segment of the rigid category exhibited a very poor performance, with a rolling resistance of 7.6 kg/tonne. More specifically, the worst segment (i.e., 7.6 kg/tonne) and the best segment (i.e., 5.6 kg/tonne) along the tested course on Highway 20 were both rigid pavements only separated by a viaduct, meaning the tire operating conditions were practically identical. This suggests again that pavement characteristics other than the pavement type are affecting the rolling resistance of heavy vehicles.

**Figure 13:**
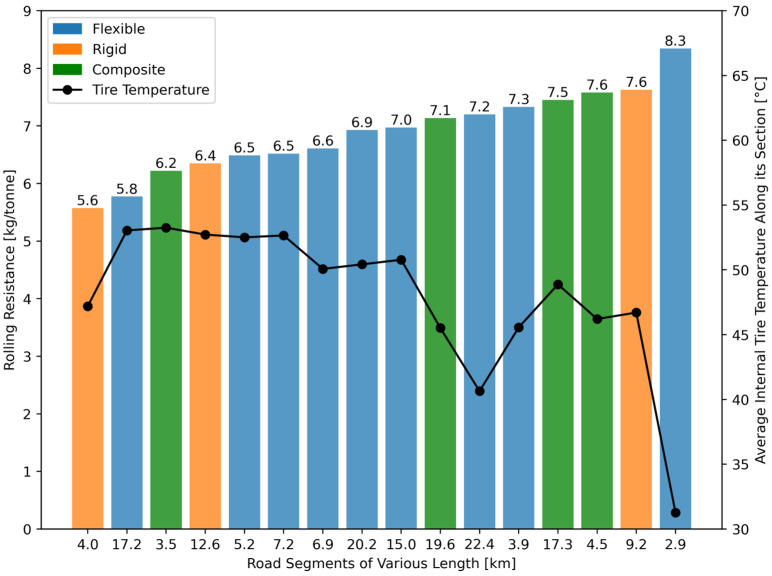
Rolling resistance coefficients measured and arranged in ascending order by pavement type, and average tire temperature for all road segments during the experimental measurements of 4 October 2023 under highway conditions at a constant vehicle speed of 95 km/h.

Since all rigid pavements in the current study have joints spaced by a regular distance, it was hypothesized that the rolling resistance of heavy vehicles circulating on rigid pavements could be linked to the state of the cement concrete slabs. The next section presents a discussion of this hypothesis. The performance of each road segment in Table 5 is presented in ascendant order in Figure 13.

### 3.7. Rigid Pavement—Slab Interaction

A detailed analysis has been performed on road segments 5 and 6, which are both rigid pavements geographically adjacent. As mentioned previously, they are also the two road segments with the greatest difference in terms of rolling resistance at equivalent tire operating conditions. The purpose of this section is to better visualize and investigate the root cause of this performance difference.

**Figure 14:**
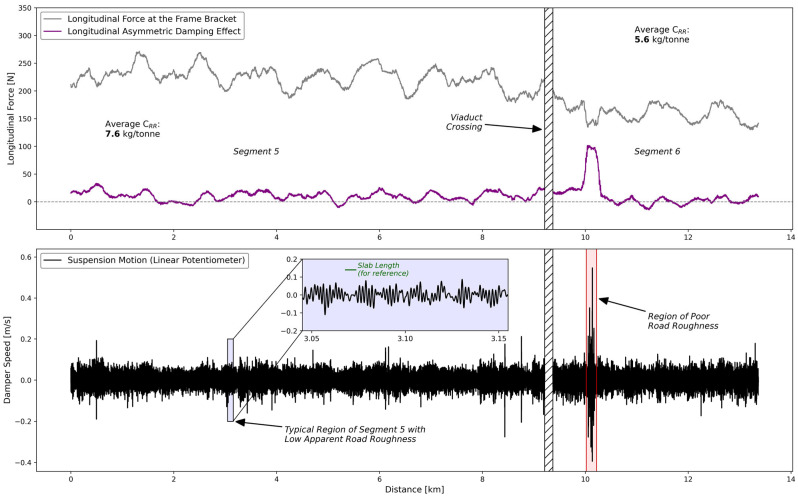
(**Top**) Measured longitudinal forces across two consecutive rigid pavements of segments 5 and 6; (**Bottom**) Damper speed and identification of typical and poor road roughness regions.

Figure 14 presents the measured longitudinal force acting on a half-axle with the Longitudinal Asymmetric Damping Effect for the two road segments in question. It shows two distinct regions: one where the rolling resistance is high (i.e., 7.6 kg/tonne) and another region where the rolling resistance is much lower (i.e., 5.6 kg/tonne). This difference does not account for the higher road slope in segment 6 (i.e., 0.27°), which would slightly increase the rolling resistance difference. Despite the presence of a poor road roughness region in segment 6, highlighted in red in Figure 14 and visible in both the damper speed and force measurement signals, the reduction in rolling resistance after the viaduct crossing remains evident.

In addition, the blue inset plot in Figure 14 shows the zoomed-in damper speed signal of a typical section along road segment 5. Upon visual inspection, there appears to be a certain periodicity that could be associated with the slab length, as illustrated in green. This is relevant because road roughness is one of the pavement-related phenomena of interest, as indicated in the Sankey diagram of power consumption in Figure 1.

**Figure 15:**
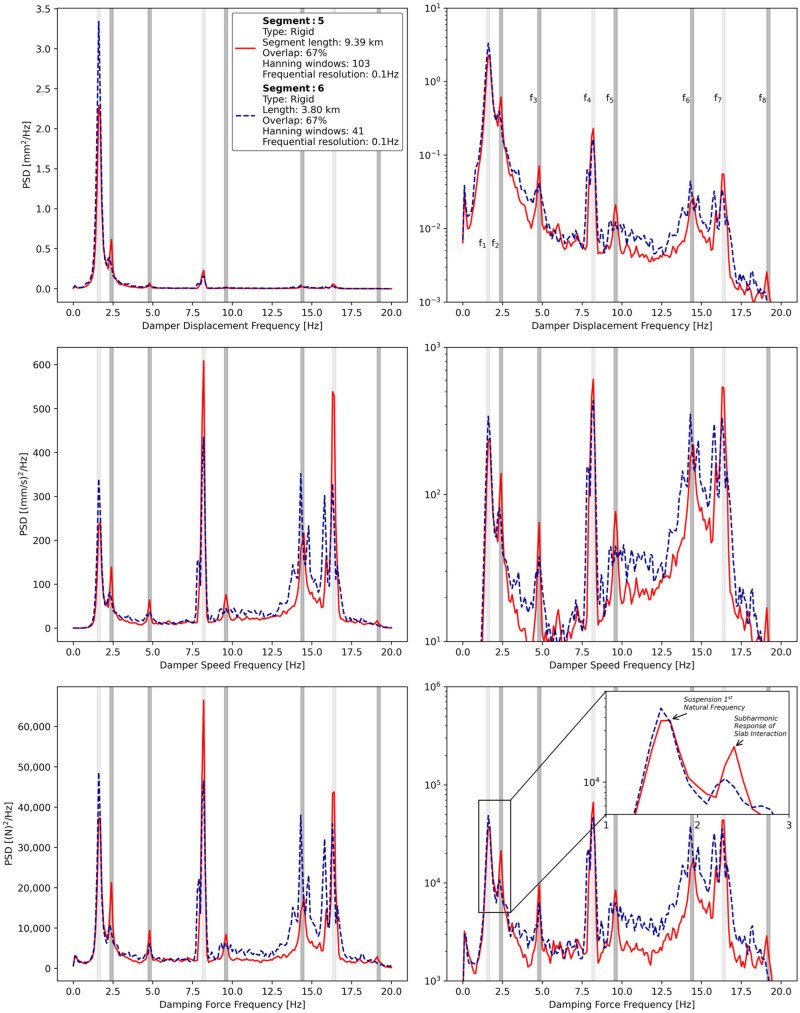
PSDs of suspension motion, speed, and damping force in linear and semi-log scales for the two consecutive rigid pavement segments (5 and 6), with the frequencies of interest (i.e., *f*_1_ to *f*_8_) defined in Table 6.

To further investigate this observation, the power spectrum densities (PSD) of suspension motion (i.e., displacement and speed) and damping force for both road segments were computed. To compare the amplitudes of frequencies related to slab presence between the two segments, the data in the red zone identified in Figure 14 was excluded from this analysis. This corresponded to only 5% of the data in segment 6. The three PSDs for both road segments are displayed in either linear or semilogarithmic scale and are presented in Figure 15.

The PSD plots show peaks at certain frequencies (e.g., from *f*_1_ to *f*_8_) that are either common to every road segment (i.e., lighter rectangles) or different between rigid road segments (i.e., darker rectangles). As indicated in the inset plot of Figure 15, the frequency *f*_1_ corresponds to the first natural frequency of the pneumatic suspension, which aligns with both expectations and the manufacturer’s specifications [62]. This frequency is common to both road segments, as shown in Figure 15, where the amplitude is higher in segment 6. This is probably because segment 5 has, on average, a lower road roughness across all wavelengths (from 0.5 m to 50 m) that can dynamically interact with the suspension [8].

Other frequencies that are common to both road segments are 8.2 Hz (i.e., *f*_4_) and 16.4 Hz (i.e., *f*_7_). The origin of these frequencies can be understood from the vehicle speed (i.e., V ≅ 95 km/h) and the effective radius of the wheels (i.e., r ≈ 0.51 m):

(19) $$\frac{V}{r}=\frac{95\;[km/h]}{0.51\;[m/rad]}\cdot \frac{1[m/s]}{3.6\;[km/h]}\cdot \frac{1\;[Hz]}{2\pi \;[rad]}≅8.2\;[Hz]$$

The frequency at 8.2 Hz is hypothesized to be linked to wheel imbalance and tire rotational vibration [63]. Data from the various transducers installed on the suspension suggest that the axle undergoes axial translational oscillations at the same frequency as the wheels. This oscillation is observable in the force measurements at the frame bracket, as well as in both the suspension motion and damping force measurements, likely because the dampers appear to be intentionally designed with a slight vertical misalignment to account for this specific oscillatory behaviour. However, modelling this phenomenon falls beyond the scope of the current study. Furthermore, the secondary peak observed in Figure 15 for segment 6, around 8 Hz, is likely caused by slight vehicle speed fluctuations immediately following the viaduct crossing. This interpretation is based on the fact that the integral of the PSDs around this peak yields the same value in both cases.

Additionally, the second harmonic of this phenomenon (i.e., 16.4 Hz) was also observed. The wheel rotation and its second harmonic were detectable across all road segments, indicating that this behaviour is not inherently related to the pavement. To the best of the authors’ knowledge, these tire-related aspects are not typically captured or considered in rolling resistance measurements conducted under laboratory conditions. Thus, it is not possible to draw definitive conclusions about the potential impact of these frequencies on rolling resistance.

The frequencies *f*_2_, *f*_3_, *f*_5_, *f*_6_, and *f*_8_ (i.e., darker rectangles in Figure 14) were clearly present for segment 5, but were only weakly visible for segment 6. The rigid pavements on the tested course were all made of 5.5 m long jointed slabs. From this information, it becomes apparent that the frequency *f*_3_ can be obtained as follows:

(20) $$\frac{95\;[km/h]}{5.5\;[m/cycle]}\cdot \frac{1\;[m/s]}{3.6\;[km/h]}≅4.8\;[Hz]$$

Based on the data from Figure 14, it is hypothesized that a dynamic interaction occurred between the heavy vehicle suspension and the slabs of jointed rigid pavements, referred to as the “slab interaction”. Subsequently, the frequencies *f*_5_, *f*_6_, and *f*_8_ (i.e., 9.6, 14.4, and 19.2Hz, respectively) would correspond to the harmonics of the fundamental frequency f_3_ (i.e., 4.8 Hz). It should be specified that the vehicle’s second natural frequency has been identified as a broad damped bump located around 14 Hz. This indicates that the dynamics of the vehicle are juxtaposed with the third harmonic of slab interaction.

In addition, the frequency *f*_2_ (i.e., 2.4 Hz), being exactly half of the fundamental frequency (i.e., 4.8 Hz), would correspond to a subharmonic response of the system. Two possible explanations for this have been identified:

- Nonlinearities in the suspension system, specifically in the tires and air bellows [64], could be the cause of subharmonic vibrations. This can persist in systems subjected to periodic loads, even in the presence of stochastic dynamics [65].
- The deterioration of the slabs on the tested rigid pavements could lead to subharmonic responses in the vehicle suspension. This could be related to faults at the joints, where uneven slab alignment increases the probability of the suspension being excited at a frequency corresponding to two slabs. Inconsistent stiffness in the subbase could amplify this effect [66,67].

The suspension characteristics remained unchanged between the two road segments, yet the amplitude of the first natural frequency for segment 6 is higher than that of segment 5, despite segment 5 exhibiting a much more pronounced subharmonic response. For these reasons, it is believed by the authors that the second explanation mentioned above is the dominant one.

From this idea, it is hypothesized that deteriorated slabs of jointed rigid pavements may increase the rolling resistance of heavy vehicles. This increase appears to be reflected in the suspension motion. Moreover, it remains uncertain whether current experimental methods like the falling weight deflectometer and the rolling wheel deflectometer, typically used for assessing load transfer efficiency, are capable of detecting the same effect [55,68]. Table 6 summarizes the frequency of interest in the PSDs in Figure 15.

To the best of the authors’ knowledge, only one experimental study in the literature, using engine control unit data and accelerometers, has observed the slab interaction directly on jointed rigid pavements and suggested a link with rolling resistance [54]. On the other hand, theoretical studies in the literature either did not consider slab interaction [69] or modelled the load transfer efficiency between consecutive slabs and concluded that its impact was practically negligible [70,71].

In addition, some road agencies have used skewed, randomly spaced joints to eliminate harmonic-induced ride quality problems caused by faults [66], but to the best of the authors’ knowledge, this concept has never been addressed from the perspective of rolling resistance. These elements suggest a discrepancy between what is understood from theoretical modelling and what is measured under real driving conditions. The data behind Figure 14 and Figure 15 indicates that more experimental research is needed on this topic.

## 4. Conclusions

This study highlights the critical role of pavements in influencing rolling resistance under real-world driving conditions, revealing significant variability both within and across pavement types. Composite pavements exhibited the worst performance, with rolling resistance averaging 7% higher than flexible pavements, likely due to interactions between bituminous top layers and older cement-based foundations under heavy loads. However, the variability within a single pavement category was more pronounced than the variability between categories. For instance, two rigid pavements separated by a viaduct showed a 34% difference in rolling resistance, potentially linked to slab deterioration and subharmonic vehicle responses.

Key findings and recommendations include the following:

- Pavement type alone cannot fully explain rolling resistance variations. Future analyses should include factors like pavement age, macrotexture, and layer thickness.
- Tire internal temperature significantly impacts rolling resistance, with a reduction of 0.09 kg/tonne observed for every 1 °C increase in tire temperature. These findings highlight the limitations of laboratory-based methods, which fail to account for real-world factors such as vehicle speed, axle load, and ambient temperature [32,49].
- The study relied on measurements taken on a single day. To ensure the validity and generalizability of the results, future research should encompass a broader range of meteorological conditions.
- Improving rolling resistance computations by integrating accelerometer/gyroscope data could mitigate variability caused by road slope and provide more precise measurements.
- Vehicle speed was maintained at a constant 95 km/h in this study to focus on steady-state highway conditions. However, this may not accurately reflect conditions typically encountered in urban environments.

These results emphasize the need for further investigations to bridge the gap between laboratory findings and real-world observations. Such efforts would support the development of more efficient pavement and tire designs, encouraging greater adoption of low-rolling resistance technologies.

## Figures and Tables

**Table 1:** Summary of various experimental studies evaluating the rolling resistance of pavements with their corresponding level of measurements.

Experimental Method	Level	Comments	References
Coast-down tests	N/A	Not in normal driving conditions	[5]
Vehicle operational data	1	- Encompasses all factors influencing power consumption - Trade-off between accuracy and comprehensiveness of the model	[3,4,11,12,13,14,15,16,17,18]
Driving torque measurements	2	- Encompasses all aspects of vehicle dynamics - Trade-off between sensitivity and fatigue life of the transducers	[19,20]
Instrumented axle	3	Found not suitable for rolling resistance measurements	[21]
Instrumented trailer	3	Currently exists only for passenger car tires, which cannot capture the structure-induced rolling resistance (SRR) of heavy vehicles	[22,23,24,25]
Traffic Speed Deflectometer (TSD)	4	Only useful for SRR measurements, hence not capturing the pavement macrotexture and roughness	[26,27]

**Table 2:** Number of segments and total length of each pavement type on which the measurement system collected data on 4 October 2023 on Highway 20 in Quebec, QC, Canada.

Pavement Type	Number of Segments	Total Length [km]
Flexible	9	102.3
Composite	4	45.7
Rigid	3	26.3
Total	16	174.4

**Table 3:** List of all the identified potential sources of error when using the measurement system with embedded transducers for assessing the rolling resistance coefficient in the context of the dataset of 21 October 2023.

Symbol	Potential Source of Error	Origin	Effect on C_RR_ Uncertainty [kg/Tonne]
ε_1_	Analog-to-digital conversion	Data acquisition system	±0.033
ε_2_	Random noise	Data acquisition system	≅0
ε_3_	Difference in coefficient of thermal expansion	Strain gauges and spring element	≅0
ε_4_	Temperature change on gauge factor	Strain gauges	±0.0052
ε_5_	Temperature change on modulus of elasticity of Ti-6AL-4V	Spring element	±0.017
ε_6_	Load cells—Linearity	Spring element	≅0
ε_7_	Load cells—Hysteresis	Spring element	≅0
ε_8_	Load cells—Repeatability	Strain gauges and spring element	≅0
ε_9_	Load cells—Creep	Strain gauges and their bonding material	≅0

**Table 4:** Summary of the average values of road slope and tire temperature for each pavement type and their effect on the measured rolling resistance coefficient; a negative value indicates that rolling resistance might be underestimated in relation to a perfectly flat road and constant tire temperature, and vice versa.

Pavement Type	Tire Temperature		Road Slope		Sum of Disturbing Factors
	Average [°C]	Effect on C_RR_ ^1^ [kg/Tonne]	Average [°]	Effect on C_RR_ ^2^ [kg/Tonne]	Effect on C_RR_ ^3^ [kg/Tonne]
Flexible	48.2	0.0022	0.015	0.026	0.028
Composite	47.5	0.070	−0.070	−0.12	−0.050
Rigid	49.7	−0.13	0.010	0.017	−0.11
All pavements	48.3	N/A (same tire temperature)	−0.0079	−0.014	N/A

^1^ This was calculated with the deviation from the average tire temperature of 48.3 °C and the coefficient of −0.09 kg/tonne/°C Presented in Figure 11. ^2^ This was calculated with Equation (2) presented in Section 2.1. ^3^ This was calculated by adding the effect of road slope and tire temperature.

**Table 5:** Summary of the average tire temperature, road slope, force measurements, and rolling resistance across all road segments of the dataset.

Segment Number	Pavement Type	Length	Tire Temperature	Average Road Slope	Frame Bracket ^1^	Longitudinal Asymmetric Damping Effect ^2^	Rolling Resistance ^3^	
		[km]	[°C]	[°]	[N]	[N]	[N]	[kg/Tonne]
1	Flexible	3.0	31.3	−0.42	199	57	257	8.3
2	Flexible	22.7	40.6	0.14	170	52	221	7.2
3	Flexible	3.9	45.6	0.03	189	36	225	7.3
4	Composite	4.6	46.2	−0.08	219	14	233	7.6
5	Rigid	9.4	46.7	−0.02	224	10	235	7.6
6	Rigid	4.1	47.2	0.27	161	11	171	5.6
7	Composite	17.6	48.9	−0.12	203	26	229	7.5
8	Flexible	7.0	50.1	−0.03	193	10	203	6.6
9	Flexible	15.3	50.8	0.02	194	20	214	7.0
10	Composite	20.0	45.5	0.02	215	4	219	7.1
11	Flexible	20.5	50.4	0.08	209	4	213	6.9
12	Flexible	5.2	52.5	−0.13	185	14	299	6.5
13	Flexible	17.4	53.0	−0.01	172	6	178	5.8
14	Composite	3.5	53.3	−0.31	185	6	191	6.2
15	Rigid	12.9	52.7	−0.05	171	25	195	6.4
16	Flexible	7.3	52.7	−0.19	176	24	200	6.5

^1^ As explained in Section 2.2, this is the measured value [$avg(F_{BL})$] obtained using the load cells installed at the frame bracket. ^2^ As explained in Section 2.3, this is the measured value [$avg\left(F_{D}\right)\cos\left(\phi \right)$] obtained using the load pin installed at the upper pivot of the damper. ^3^ This is the sum of the frame bracket longitudinal force and the Longitudinal Asymmetric Damping Effect.

**Table 6:** Frequencies of interest obtained by computing the power spectrum density of suspension motion and damping force on the jointed rigid pavements of road segments 5 and 6.

Label of the Frequency	Value [Hz]	Physical Interpretation	Mathematical Interpretation
*f*_1_	1.6	Suspension characteristics	Suspension natural frequency
*f*_2_	2.4	Two-slab distance	Subharmonic of slab interaction
*f*_3_	4.8	One-Slab distance	Fundamental frequency of slab interaction
*f*_4_	8.2	Rotation of wheels	Fundamental frequency of wheel rotation
*f*_5_	9.6	Half-slab Distance	Second harmonic of slab interaction
*f*_6_	14.4	Third-Slab Distance	Third harmonic of slab interaction (Confounded with the second natural frequency of the vehicle that is around 14 Hz)
*f*_7_	16.4	Twice the Rotation of wheels	Second harmonic of wheel rotation
*f*_8_	19.2	Quarter-Slab Distance	Fourth harmonic of slab interaction

## Data Availability

The original contributions presented in the study are included in the article material, further inquiries can be directed to the corresponding author/s.
